# Solitary Nitric Oxide Signaling Mediates Mild Stress-Induced Anxiety and Norepinephrine Release in the Bed Nucleus of the Stria Terminalis during Protracted Ethanol Withdrawal

**DOI:** 10.1155/2021/2149371

**Published:** 2021-11-29

**Authors:** Zhenglin Zhao, Sang Chan Kim, Yu Jiao, Yefu Wang, Bong Hyo Lee, Hee Young Kim, Chul Won Lee, Chae Ha Yang, Rongjie Zhao

**Affiliations:** ^1^Department of Psychopharmacology, Qiqihar Medical University, Qiqihar 161006, China; ^2^Medical Research Center, College of Korean Medicine, Daegu Haany University, Gyeongsan 38610, Republic of Korea

## Abstract

Ethanol withdrawal (EtOHW) alters the pattern of neurohormonal and behavioral response toward internal and external stimuli, which mediates relapse to alcohol use even after a long period of abstinence. Increased noradrenergic signaling from the nucleus tractus solitarius (NTS) to the bed nucleus of the stria terminalis (BNST) during EtOHW underlies withdrawal-induced anxiety, while nitric oxide synthase (NOS) inhibitors injected into the periaqueductal area attenuate EtOHW-induced anxiety. Therefore, this study investigated the involvement of NOS within the NTS in anxiety and increased norepinephrine (NE) release in the BNST during protracted EtOHW in rats exposed to a mild stress. Rats were intraperitoneally administered 3 g/kg/day EtOH for 21 days followed by 28 days of withdrawal, and on the 28^th^ day of withdrawal, the rats were subjected to restraint stress for 7 minutes. The elevated plus maze test was employed to evaluate anxiety-like behavior in rats, and in vivo microdialysis was used to measure the extracellular NE level in the BNST. In elevated plus maze tests, EtOHW rats but not EtOH-naive rats exhibited anxiety-like behavior when challenged with 7-minute mild restraint stress, which was, respectively, mitigated by prior intra-NTS infusion of the nitric oxide scavenger 2-(4-carboxyphenyl)-4,4,5,5-tetramethylimidazoline-1-oxyl-3-oxide (carboxy-PTIO), nonselective NOS inhibitor NG-nitro-L-arginine methyl ester (L-NAME), or selective neuronal NOS (nNOS) inhibitor 7-nitroindazole (7-NI). Each of these agents also decreased the plasma corticosterone levels in EtOHW rats. In in vivo microdialysis, prior intra-NTS infusion of carboxy-PTIO, L-NAME, or 7-NI attenuated the mild stress-induced NE release in the BNST of EtOHW rats. Additionally, EtOHW rats showed increased solitary nNOS gene and protein expression. Moreover, the anxiolytic effect of intra-NTS administration of 7-NI was abolished by subsequent intra-NTS administration of sodium nitroprusside. These results suggest that elevation of solitary nitric oxide signaling derived from nNOS mediates stress-precipitated anxiety and norepinephrine release in the BNST during protracted EtOHW.

## 1. Introduction

Relapse is a major barrier in alcoholism therapy [1]. Ethanol withdrawal (EtOHW) leads to adaptation of neurotransmission in the brain that often persists long after complete remission of EtOHW symptoms. This adaptation sensitizes physiological and behavioral responses to internal and external stimuli, characterized by exacerbated pathophysiological responses toward mild stress or even nonstress stimuli [2]. This phenomenon during EtOHW is a stress sensitization that mimics the allodynia in pain medicine in which innocuous stimuli provoke a painful sensation, resulting in relapse even after a long period of abstinence [3].

Elevated anxiety during EtOHW is the major negative emotional component for alcoholism relapse [4], and several lines of evidence indicate that a heightened norepinephrine (NE) signaling in the bed nucleus of the stria terminalis (BNST) is responsible for it. The BNST is a component of the extended amygdala; NE signaling in the BNST along with corticotrophin releasing factor (CRF) is the key factor mediating the anxiety associated with withdrawal of drugs of abuse [5]. NE triggers the extracellular release of CRF, and EtOHW increases the extracellular NE level in the central nucleus of the amygdala (another component of the extended amygdala) [6] and the CRF level in the BNST [7], which underlie EtOHW-induced anxiety. EtOH and morphine share many pharmacological similarities including induction of extracellular NE release in the BNST [8], and extracellular NE release is increased in the BNST in both spontaneous and naloxone-precipitated morphine withdrawal [9, 10]. Therefore, the extracellular NE level may be enhanced in the BNST during EtOHW in association with elevated anxiety. Moreover, Valdez et al. [11] reported that acute mild-restraint stress (AMRS) produced significant anxiety in rats during protracted EtOHW but not in their EtOH-naive counterparts, indicating an allodynia-like phenomenon. Taken together, these observations suggest that AMRS may induce sensitized NE release in the BNST, which is associated with anxiety in rats during protracted EtOHW.

The BNST is a complex consisting of multiple nuclei, broadly divided into anterior and posterior, dorsal and ventral, and medial and lateral parts, and it is heavily innervated by noradrenergic projections arising from both A1/A2 in the nucleus tractus solitarius (NTS) and A6 (locus coeruleus) [12]. However, immunocytochemical and retrograde tracer analyses indicated that the noradrenergic inputs to the ventral BNST (vBNST) are derived mainly from the NTS-A2 [13, 14] and are critical for the negative emotions induced by withdrawal of drugs of abuse. For example, opiate withdrawal precipitated by opioid receptor antagonists was shown to increase NE release in the vBNST and to enhance c-Fos expression in both the vBNST and vBNST-projecting NTS-A2 neurons, whereas intra-vBNST infusion of *β*-adrenergic antagonists reduced the withdrawal-associated conditioned place aversion [14–16]. Neurochemical lesions of the noradrenergic bundle originating from the NTS-A2, but not from A6, attenuated the withdrawal aversion [14]. These observations suggest increased activity in the NTS-vBNST noradrenergic pathway during withdrawal of drugs of abuse, which is attributed to activation of NTS-A2 neurons. A wide range of neurotransmitters and neuropeptides, including glutamate, gamma-aminobutyric acid (GABA), nitric oxide (NO), galanin, and neuropeptide Y, are present in the NTS-A2 and regulate NE neuronal activities [17]. It is well documented that NO synthase (NOS) inhibiters have anxiolytic effects [18–20]; moreover, the NO system plays an important role in EtOHW anxiety and NE release induced by drug abuse. Bonassoli et al. [21] reported that EtOHW activated NO-producing neurons in the brainstem, while local infusion of NOS inhibitors into the brainstem regions such as the periaqueductal gray matter area and the dorsal raphe nucleus mitigates EtOHW anxiety [22, 23]. Our previous studies indicated that acute nicotine induces NE release in the hypothalamus and amygdala via the NTS NO pathway [24, 25]. These findings suggest that the NTS NO system may mediate the enhanced NTS-vBNST noradrenergic activities during protracted EtOHW.

Taken together, all the above observations lead to speculation that there may be abnormally heightened NTS-vBNST NE neuronal activities mediated by the NTS NO system during protracted EtOHW, which underlie anxiety in response to challenge with otherwise innocuous stimuli. To test this, in the present study, rats were exposed to a 7-minute AMRS during protracted EtOHW, and the effects of intra-NTS infusion of NOS inhibitors on anxiety-like behavior and NE release in the vBNST of EtOHW rats were examined.

## 2. Materials and Methods

### 2.1. Animals and Surgery

Eight-week-old male Sprague–Dawley rats (250-270 g) were provided by the Laboratory Animal Center at Qiqihar Medical University (Qiqihar, China) and housed in individual cages under standardized conditions (12 : 12 hour light/dark cycle, 21–23°C, free access to food and water). All rats were randomly assigned to the various experimental groups. All experimental procedures were approved by the Animal Care and Use Committee of Qiqihar Medical University (approval number: QMUAECC-2016-28) and were performed adhering to the National Institutes of Health guidelines.

After 7 days of acclimatization, the rats were stereotactically (Kopf Instruments, Tujunga, CA) implanted with bilateral microinjection guide cannulae targeting the NTS and/or a unilateral microdialysis probe guide cannula targeting the right ventral vBNST under sodium pentobarbital (50 mg/kg, intraperitoneally) anesthesia. In brief, after fixing the rat on the instrument, a longitudinal midline incision was made on the shaved and disinfected scalp, creating the surgical window by exposing the bregma and the lambda and clearing the skull surface using sterile cotton-tips dipped in 3% H_2_O_2_ solution. The coordinates of the bregma and the lambda were used to level the skull and act as the references, and the hydrogen peroxide solution (also a disinfectant) was used to eliminate the soft tissues to prevent a possible infection afterward. After the confirmation of a horizontally levelled skull, three small holes were drilled on the skull, respectively, targeting the right ventral vBNST and both sides of the NTS according to their coordinates, and guide cannulae were implanted through the holes and secured with two small screws and dental cement. The coordinates of the NTS and vBNST relative to bregma, according to the atlas of Paxinos and Watson [26], were as follows: anterior–posterior, −13.6 and−0.3 mm; medial–lateral, 0.8 and 1.4 mm; and dorsal ventral, 6.5 and 7.5 mm, respectively. The microinjection guide cannulae were positioned 1.5 mm above the targets. During the surgery, a small amount of lubricant eye ointment was applied to each rat to prevent corneal damage, and antibiotics and pain killers were also used for the postsurgery care. At the end of each experiment, the rats were euthanized and decapitated, the brain was extracted and stored at -80°C, and then coronal cryotome sections were made to observe the probe and injection positions, and if more accurate histological confirmation was desired, a Coomassie blue staining was used; finally, the rats with the correct targets were included for statistical analysis.

### 2.2. Experimental Protocols and Collection of Blood and NTS Tissues

**Figure 1:**
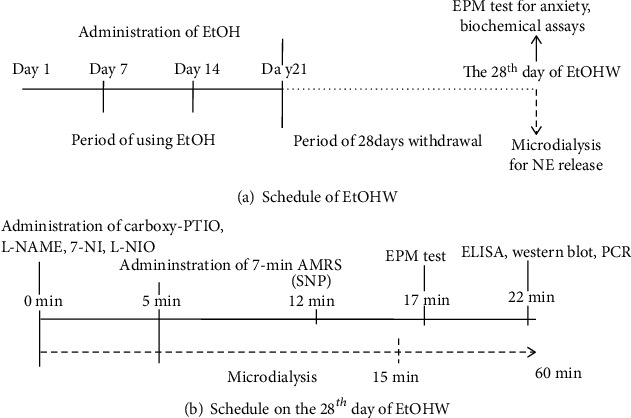
(a) Time schedules for EtOHW and (b) the 28^th^ day of EtOHW.

After a 7-day recovery period after the surgery, the rats were intraperitoneally administered 3 g/kg/day EtOH (20% w/v) or saline for 21 days followed by 28 days of withdrawal. On the 28th day of EtOHW or saline withdrawal, the rats received 7-minute AMRS in a 6.4-cm-diameter plastic cylinder constructed from a 500 mL mineral water bottle and underwent in vivo microdialysis or a behavioral test in an elevated plus maze (EPM) (Figure 1).

Immediately after the EPM test, the rats were euthanized and decapitated. Trunk blood was collected to measure plasma corticosterone (CORT) levels. The entire brain was removed and stored at −80°C, and NTS tissues were obtained by the punch-out technique [25] according to the coordinates outlined above for Western blot analysis.

### 2.3. Intra-NTS Microinfusion

To examine the possible effects of NOS inhibitors on AMRS-induced anxiety and NE release in the vBNST during protracted EtOHW, bilateral intra-NTS microinfusion of the following agents was performed: NO scavenger 2-(4-carboxyphenyl)-4,4,5,5-tetramethylimidazoline-1-oxyl-3-oxide (carboxy-PTIO; 0.2 nmol/100 nL each side; Sigma-Aldrich, St. Louis, MO), nonselective NOS inhibitor NG-nitro-L-arginine methyl ester (L-NAME; 30 nmol/100 nL each side; Sigma-Aldrich), selective neuronal NOS (nNOS) inhibitor 7-nitroindazole (7-NI; 30 nmol/100 nL each side; Sigma-Aldrich), selective endothelial NOS (eNOS) inhibitor L-N5-(1-iminoethyl)ornithine dihydrochloride (L-NIO; 3 nmol/100 nL each side, Sigma-Aldrich), and NO donor sodium nitroprusside (SNP; 0.1 nmol/100 nL each side; Sigma-Aldrich). The agents were infused using a 30-gauge injector (1.5 mm longer than the guide cannula) through the intra-NTS guide cannulae (22-gauge) using a microinjection pump for 45 seconds. All drugs were dissolved in modified Ringer's solution containing 150 mM NaCl, 3.0 mM KCl, 1.4 mM CaCl_2_, and 0.8 mM MgCl_2_ in 10 mM phosphate buffer, pH 7.2. All drugs were introduced 5 minutes before AMRS except for SNP, which was given after intra-NTS administration of 7-NI, immediately after AMRS, to examine the effect of subsequent intra-NTS administration of SNP on the anxiolytic action of 7-NI. The rats were subjected to the EPM test 5 minutes after AMRS (Figure 1).

### 2.4. EPM Test

On day 28 of EtOHW, after treatment with drugs and exposure to AMRS, the rats were examined in the EPM to measure anxiety-like behaviors as described previously [6]. Briefly, the EPM was composed of two closed arms (50 cm long × 10 cm wide, with black walls 40 cm high) and two open arms (without walls), which were arranged perpendicularly, elevated above the ground and video recorded using a video tracking system (Shanghai Xinruan Technology Co., Shanghai, China). During the examination, each rat was placed in the center of the EPM, and the number of entries into the arms and the time spent in each arm were monitored for 5 minutes. The percentages of number of entries into the open arms and time spent in the open arms, relative to the total entries/time, were calculated as follows: %Entries_into open arms_ = Entries_into open arms_/(Entries_into open arms_ + Entries_into closed arms_) × 100%and%Time_spent in open arms_ = Time_spent in open arms_/(Time_spent in open arms_ + Time_spent in closed arms_) × 100%.

### 2.5. Enzyme-Linked Immunosorbent Assay (ELISA)

Blood samples (1 mL from each rat) collected in chilled microcentrifuge tubes containing 20 *μ*L EDTA (20 mg/mL) were centrifuged for 10 minutes at 1,500 × *g* and 4°C to separate the plasma. Plasma CORT levels were determined using a commercial ELISA kit (Abcam, Cambridge, UK) in accordance with the manufacturer's instructions, and values are presented as nanograms per milliliter.

### 2.6. Measurement of Extracellular NE

On day 28 of EtOHW, a microdialysis probe (CMA11, 2-mm membrane length, 6,000 Da; Carnegie Medicine, Stockholm, Sweden) was inserted into the vBNST via the guide cannula. The probe was constantly perfused (1.5 *μ*L/min) with Krebs–Ringer buffer (147 mM NaCl, 3.4 mM CaCl_2_, and 4.0 mM KCl in polished water) containing 5 *μ*M nomifensine using a microinjection pump for 2 hours. Next, microdialysates were collected at 15-minute intervals in microcentrifuge tubes containing 1 *μ*L 5% perchloric acid, and basal NE levels were determined by measuring three consecutive dialysates prior to experimental administration. NE levels in the microdialysates were measured by injecting a 15 *μ*L dialysate into a high-performance liquid chromatography system equipped with a coulometric detector (Coulochem II; ESA Laboratories, Bedford, MA) [25]. After measuring the basal NE level, the rats received bilateral intra-NTS injection of carboxy-PTIO, L-NAME, 7-NI, or L-NIO. Five minutes after injection of the drugs, the rats were subjected to AMRS. Microdialysates were continuously collected during these experimental processes and thereafter (60 minutes total) and analyzed for NE levels (Figure 1).

### 2.7. Western Blot Analysis

The whole NTS tissues from each rat that underwent the designated experimental schedule were homogenized in lysis buffer containing 20 mM Tris, 5 mM EDTA, 1% Nonidet P-40 (v/v), and phosphatase and protease inhibitors. The homogenates were centrifuged for 20 minutes at 16,000 × *g* and 4°C, and the total protein content in each supernatant was quantified by bicinchoninic acid assay. The proteins were separated by 8% sodium dodecyl sulfate polyacrylamide gel electrophoresis and transferred onto polyvinylidene difluoride membranes (Millipore, Bedford, MA). The proteins in the membrane were reacted with the following primary antibodies: rabbit polyclonal antibodies to nNOS, eNOS, phospho-nNOS Ser1417, *β*-actin (all from Abcam), and phospho-eNOS Ser1177 (Cell Signaling Technology, Beverly, MA). The membranes were then reacted with a peroxidase-conjugated anti-rabbit secondary antibody (Cell Signaling Technology). Finally, the bands of the proteins of interest were visualized by enhanced chemiluminescence (Amersham Biosciences, Piscataway, NJ), densitometrically analyzed with the aid of ImageJ, and the relative expressions of the proteins were calculated as follows (the *β*-actin was used as the loading control): %of protein expression relative to the control group = (density of the designated protein/Density of *β* − actin)_treated_/(density of the designated protein/density of *β* − actin)_control_ × 100%.

### 2.8. Real-Time RT-PCR

Total RNA was extracted from the whole NTS tissues of each rat that underwent the designated experimental schedule using TRIzol Reagent (Invitrogen, Carlsbad, CA) and reverse transcribed into cDNA using a reverse-transcription PCR kit (TaKaRa Bio Inc., Shiga, Japan). Real-time PCR amplification was performed on the CFX-96 PCR system (Bio-Rad, Hercules, CA) using SYBR green premix (TaKaRa) in accordance with the manufacturer's protocol. The following primers were synthesized by Beijing Liuhe BGI Gene, Co. (Beijing, China): nNOS, 5′-TCCCTCTAGCCAAAGAATTTCTCG-3′ (forward) and 5′-GGTAGGTGCTGGTGCTTTCAA-3′ (reverse); *β*-actin, 5-GTCGTACCACTGGCATTGTG-3 (forward) and 5-GCCATCTCTTGCTCGAAGTC-3 (reverse). The housekeeping gene *β*-actin was used to normalize the gene expression measurements, and the relative level of specific mRNA was calculated using the following formula and presented as 2^−*ΔΔ*CT^: ΔCT = CT_nNOS_ − CT_*β*−actin_, ΔΔCT = ΔCT_EtOH_ − ΔCT_Saline_.

### 2.9. Statistical Analysis

All data are presented as the mean ± SEM. Two-way analysis of variance (ANOVA) with Bonferroni posthoc test, or one-way ANOVA followed by Newman–Keuls post-hoc test was performed to statistically evaluate data. All analyses were performed using GraphPad Prism 7.0 (GraphPad Software, San Diego, CA), and differences with *p* values <0.05 were considered statistically significant.

## 3. Results

### 3.1. Effects of Carboxy-PTIO, L-NAME, 7-NI, and L-NIO on AMRS-Induced Anxiety during Protracted EtOHW

EtOHW produces anxiety-like behaviors in rats that disappear spontaneously after a certain time depending on a number of variables, including animal species and EtOH regimen. In preliminary experiments, we found that EtOHW rats did not display any anxiety-like behaviors in the EPM test 28 days after the final dose of a 21-day EtOH treatment, in agreement with the observation by Valdez et al. [11]. Meanwhile, 7-minute AMRS alone did not produce any anxiety-like behaviors in EtOH-naive rats. Therefore, to observe the interactive effect of EtOHW and an AMRS, in the present study, the rats were exposed to 7-minute AMRS on day 28 of EtOHW and then subjected to the EPM test.

**Figure 2:**
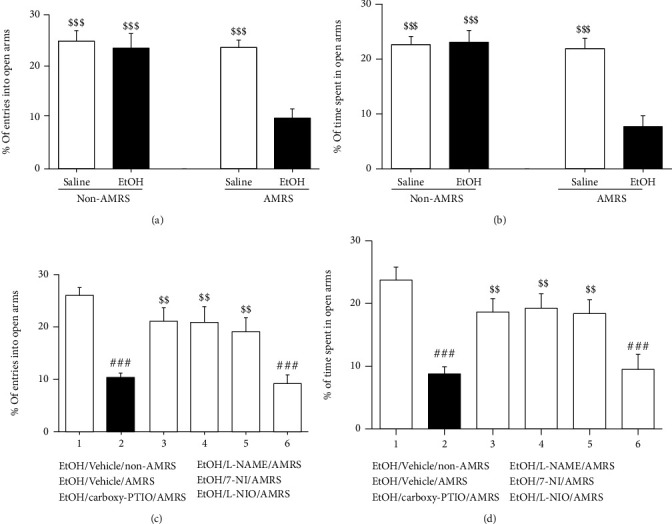
Effects of carboxy-PTIO, L-NAME, 7-NI, and L-NIO on AMRS-induced anxiety-like behavior. Data are expressed as the mean ± SEM (*n* = 8). (a, c) Percentage of numbers of entries into open arms of EPM. (b, d) Percentage of time spent in open arms.^###^*p* < 0.001 versus saline/vehicle/non-AMRS group; ^$$^*p* < 0.01, ^$$$^*p* < 0.001 versus EtOH/AMRS group or EtOH/vehicle/AMRS group.

As shown in Figures 2(a) and 2(b) (the data were analyzed by two-way ANOVA followed by Bonferroni post-hoc test), 28 days after the final dose of EtOH, the EtOHW rats did not show any significant anxiety-like behaviors; however, when exposed to 7-minute AMRS, EtOHW rats but not saline-treated controls displayed substantial anxiety-like behaviors, which were manifested by fewer visits and less time spent in the open arms [%entries_into open arms_: *F*_(drug)_ = 12.89, *p* < 0.01, *F*_(stress)_ = 12.52, *p* < 0.01, *F*_(drug × stress)_ = 8.71, *p* < 0.01; saline/non-AMRS group (*n* = 8) versus EtOH/AMRS group (*n* = 8), *p* < 0.001; EtOH/non-AMRS group versus EtOH/AMRS group, *p* < 0.001; saline/AMRS group (*n* = 8) versus EtOH/AMRS group, *p* < 0.001; %time_spent in open arms_: *F*_(drug)_ = 13.24, *p* < 0.01, *F*_(stress)_ = 18.09, *p* < 0.001, *F*_(drug × stress)_ = 15.01, *p* < 0.001; saline/non-AMRS group versus EtOH/AMRS group, *p* < 0.001; EtOH/non-AMRS group versus EtOH/AMRS group, *p* < 0.001; saline/AMRS group versus EtOH/AMRS group, *p* < 0.001]. However, as also seen in Figures 2(c) and 2(d) (the data analyzed by one-way ANOVA followed by Newman–Keuls posthoc test), intra-NTS infusion of carboxy-PTIO, L-NAME, or 7-NI, but not L-NIO, attenuated the anxiety-like behaviors [%entries_into open arms_: *F*_(5, 42)_ = 9.22, *p* < 0.001, EtOH/vehicle/non-AMRS group (*n* = 8) versus EtOH/vehicle/AMRS group (*n* = 8), *p* < 0.001, EtOH/vehicle/AMRS group versus EtOH/carboxy-PTIO/AMRS group (*n* = 8), *p* < 0.01; EtOH/vehicle/AMRS group versus EtOH/L-NAME/AMRS group (*n* = 8), *p* < 0.01; EtOH/vehicle/AMRS group versus EtOH/7-NI/AMRS group (*n* = 8), *p* < 0.01; EtOH/vehicle/AMRS group versus EtOH/L-NIO/AMRS group (*n* = 8), *p* > 0.05; %time_spent in open arms_: *F*_(5, 42)_ = 8.05, *p* < 0.001, EtOH/vehicle/non-AMRS group versus EtOH/vehicle/AMRS group, *p* < 0.001, EtOH/vehicle/AMRS group versus EtOH/carboxy-PTIO/AMRS group, *p* < 0.01; EtOH/vehicle/AMRS group versus EtOH/L-NAME/AMRS group, *p* < 0.01; EtOH/vehicle/AMRS group versus EtOH/7-NI/AMRS group, *p* < 0.01; EtOH/vehicle/AMRS group versus EtOH/L-NIO/AMRS group, *p* > 0.05]. Moreover, at the same dose, intra-NTS 7-NI alone did not produce any significant behavioral changes in rats (data not shown).

### 3.2. Effects of Carboxy-PTIO, L-NAME, 7-NI, and L-NIO on AMRS-Induced Plasma CORT Secretion

**Figure 3:**
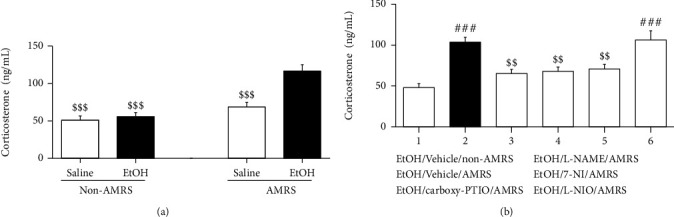
Effects of carboxy-PTIO, L-NAME, 7-NI, and L-NIO on AMRS-induced plasma CORT secretion. Data are expressed as mean ± SEM (*n* = 6). ^###^*p* < 0.001 versus saline/vehicle/non-AMRS group; ^$$$^*p* < 0.001 versus EtOH/AMRS group or EtOH/vehicle/AMRS group.

Consistent with the results of the behavioral, as seen in Figure 3(a), AMRS increased the plasma CORT level in EtOHW rats but not in saline-treated control rats [*F*_(drug)_ = 19.92, *p* < 0.001, *F*_(stress)_ = 32.25, *p* < 0.001, *F*_(drug × stress)_ = 13.96, *p* < 0.01; saline/non-AMRS group (*n* = 6) versus EtOH/AMRS group (*n* = 6), *p* < 0.001; EtOH/non-AMRS group (*n* = 6) versus EtOH/AMRS group, *p* < 0.001; saline/AMRS group versus EtOH/AMRS group, *p* < 0.001]. However, as shown in Figure 3(b), intra-NTS infusion of carboxy-PTIO [*F*_(5, 30)_ = 11.71, *p* < 0.001; EtOH/vehicle/non-AMRS group (*n* = 6) versus EtOH/vehicle/AMRS group (*n* = 6), *p* < 0.001, EtOH/vehicle/AMRS group versus EtOH/carboxy-PTIO/AMRS group (*n* = 6), *p* < 0.01], L-NAME [EtOH/vehicle/AMRS group versus EtOH/L-NAME/AMRS group (*n* = 6), *p* < 0.01], or 7-NI [EtOH/vehicle/AMRS group versus EtOH/7-NI/AMRS group (*n* = 6), *p* < 0.01], but not L-NIO [EtOH/vehicle/AMRS group versus EtOH/L-NIO/AMRS group (*n* = 6), *p* > 0.05], inhibited the increased CORT secretion.

### 3.3. Effects of Carboxy-PTIO, L-NAME, 7-NI, and L-NIO on AMRS-Induced NE Release in the vBNST

**Figure 4:**
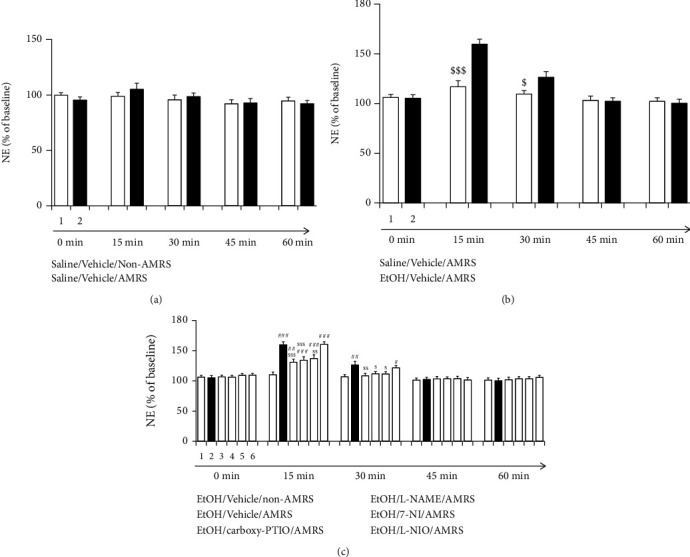
Effects of carboxy-PTIO, L-NAME, 7-NI, and L-NIO on AMRS-induced NE release in the vBNST. Data are expressed as the mean ± SEM (*n* = 7) of the percentage of the baseline. ^#^*p* < 0.05, ^##^*p* < 0.01, ^###^*p* < 0.001 versus EtOH/vehicle/non-AMRS group; ^$^*p* < 0.05, ^$$^*p* < 0.01, ^$$$^*p* < 0.001 versus EtOH/vehicle/AMRS group.

No significant differences in the basal extracellular NE level in the vBNST were found between the groups [saline/vehicle/non-AMRS group (*n* = 7): 4.25 ± 0.32 (pg/15 uL); saline/vehicle/AMRS group (*n* = 7): 4.75 ± 0.48; EtOH/vehicle/non-AMRS group (*n* = 7): 4.64 ± 0.41; EtOH/vehicle/AMRS group (*n* = 7): 5.19 ± 0.44; EtOH/carboxy-PTIO/AMRS group (*n* = 7): 4.38 ± 0.37; EtOH/L-NAME/AMRS group (*n* = 7): 4.23 ± 0.38; EtOH/7-NI/AMRS group (*n* = 7): 5.25 ± 0.37; EtOH/L-NIO/AMRS group (*n* = 7): 4.81 ± 0.37]. As seen in Figures 4(a) and 4(b), two-way ANOVA and Bonferroni posthoc comparisons revealed that 7-minute AMRS significantly increased NE release in EtOHW rats but not in saline-treated control rats [in Figure 4(a), *F*_(treatment)_ = 0.07, *p* > 0.05, *F*_(time)_ = 2.25, *p* > 0.05, *F*_(treatment × time)_ = 0.70, *p* > 0.05; in Figure 4(b), *F*_(treatment)_ = 16.79, *p* < 0.001; *F*_(time)_ = 25.63, *p* < 0.001, *F*_(treatment × time)_ = 9.55, *p* < 0.001; 15 min: saline/vehicle/AMRS group versus EtOH/vehicle/AMRS group, *p* < 0.001; 30 min saline/vehicle/AMRS group versus EtOH/vehicle/AMRS group, *p* < 0.05]. However, as shown in Figure 4(c) (the data were analyzed by two-way ANOVA followed by Bonferroni posthoc test), intra-NTS infusion of carboxy-PTIO [*F*_(treatment)_ = 9.62, *p* < 0.001, *F*_(time)_ = 87.92, *p* < 0.001, *F*_(treatment × time)_ = 4.53, *p* < 0.001; 15 min: EtOH/vehicle/non-AMRS group EtOH/vehicle/AMRS group, *p* < 0.001, EtOH/vehicle/AMRS versus EtOH/carboxy-PTIO/AMRS group, *p* < 0.001; 30 min: EtOH/vehicle/non-AMRS group versus EtOH/vehicle/AMRS group, *p* < 0.01, EtOH/vehicle/AMRS group versus EtOH/carboxy-PTIO/AMRS group, *p* < 0.01], L-NAME [15 min: EtOH/vehicle/AMRS group versus EtOH/L-NAME/AMRS group, *p* < 0.001; 30 min: EtOH/vehicle/AMRS group versus EtOH/L-NAME/AMRS group, *p* < 0.05], or 7-NI [15 min: EtOH/vehicle/AMRS group versus EtOH/7-NI/AMRS group, *p* < 0.001; 30 min: EtOH/vehicle/AMRS group versus EtOH/7-NI/AMRS group, *p* < 0.05], but not L-NIO [15 min: EtOH/vehicle/AMRS group versus EtOH/L-NIO/AMRS group, *p* > 0.05; 30 min: EtOH/vehicle/AMRS group versus EtOH/L-NIO/AMRS group, *p* > 0.05], prevented these increases. In addition, intra-NTS L-NIO alone did not significantly affect NE release in naive rats (data not shown).

### 3.4. nNOS and eNOS Protein and nNOS mRNA Expressions during Protracted EtOHW

**Figure 5:**
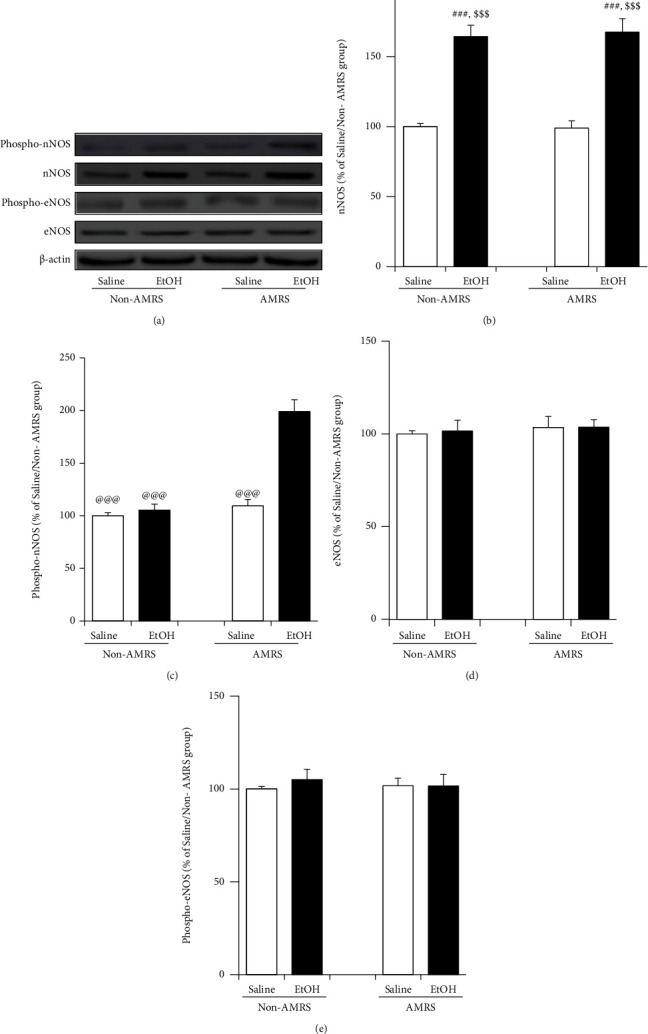
Solitary nNOS and eNOS protein expressions during EtOHW. Data are expressed as mean ± SEM (*n* = 5) of the percentage of saline/non-AMRS group.^###^*p* < 0.001 versus saline/non-AMRS group; ^$$$^*p* < 0.001 versus saline/AMRS group; ^@@@^*p* < 0.001 versus EtOH/AMRS group.

As shown in Figure 5, Western blot analyses revealed the EtOHW significantly increased the protein level of (total) nNOS in the NTS compared with saline treatment [*F*_(drug)_ = 93.69, *p* < 0.001, *F*_(stress)_ = 0.04, *p* > 0.05, *F*_(drug × stress)_ = 0.08, *p* > 0.05; saline/non-AMRS group (*n* = 5) versus EtOH/non-AMRS group (*n* = 5), *p* < 0.001; saline/AMRS group (*n* = 5) versus EtOH/AMRS group (*n* = 5), *p* < 0.001], whereas the AMRS did not alter nNOS protein expression (saline/non-AMRS group versus saline/AMRS group, *p* > 0.05; EtOH/non-AMRS group versus EtOH/AMRS group, *p* > 0.05). However, the AMRS markedly enhanced phospho-nNOS levels in EtOHW rats [F_(drug)_ = 45.06, *p* < 0.001, *F*_(stress)_ = 53.45, *p* < 0.001, *F*_(drug × stress)_ = 36.38, *p* < 0.001; EtOH/AMRS group (*n* = 5) versus EtOH/non-AMRS group (*n* = 5), *p* < 0.001; EtOH/AMRS group versus saline/AMRS group (*n* = 5), *p* < 0.001; EtOH/AMRS group versus saline/non-AMRS group (*n* = 5), *p* < 0.001] but not in saline-treated controls (saline/AMRS group versus saline/non-AMRS group, *p* > 0.05). Additionally, neither EtOHW nor AMRS significantly affected eNOS or phospho-eNOS protein expressions (*p* > 0.05 for all).

**Figure 6:**
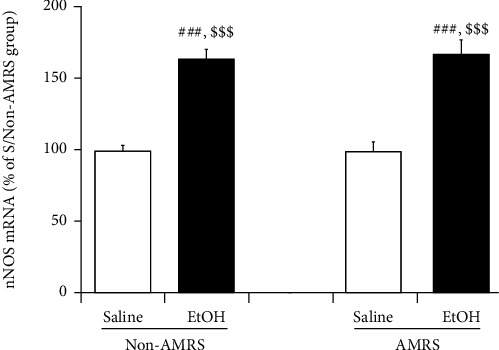
Solitary nNOS mRNA expressions during EtOHW. Data are expressed as mean ± SEM (*n* = 6) of the percentage of saline/non-AMRS group.^###^*p* < 0.001 versus saline/non-AMRS group; ^$$$^*p* < 0.001 versus saline/AMRS group.

In agreement with the Western blot data, as seen in Figure 6, PCR analysis showed enhanced nNOS mRNA expression in the NTS of EtOHW rats compared with saline-treated controls [*F*_(drug)_ = 84.40, *p* < 0.001, *F*_(stress)_ = 0.04, *p* > 0.05, *F*_(drug×stress)_ = 0.06, *p* > 0.05; saline/non-AMRS group (*n* = 6) versus EtOH/non-AMRS group (*n* = 6), *p* < 0.001; saline/AMRS group (*n* = 6) versus EtOH/AMRS group (*n* = 6), *p* < 0.001]. The AMRS did not alter nNOS mRNA expression (saline/non-AMRS group versus saline/AMRS group, *p* > 0.05; EtOH/non-AMRS group versus EtOH/AMRS group, *p* > 0.05).

### 3.5. Effects of Subsequent Intra-NTS Infusion of SNP on the Anxiolytic Action of 7-NI during EtOHW

**Figure 7:**
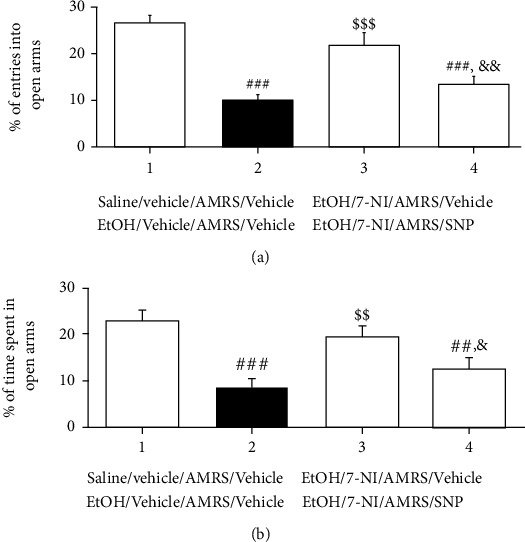
Effects of subsequent intra-NTS infusion of SNP on the anxiolytic action of 7-NI. Data are expressed as the mean ± SEM (*n* = 6). (a) Percentage of numbers of entries into open arms. (b) Percentage of time spent in open arms.^##^*p* < 0.01, ^###^*p* < 0.001 versus saline/vehicle/AMRS/vehicle group; ^$$^*p* < 0.01, ^$$$^*p* < 0.001 versus EtOH/vehicle/AMRS/vehicle group;^&^*p* < 0.05, ^&&^*p* < 0.01 versus EtOH/7-NI/AMRS/vehicle group.

To further determine the involvement of solitary nNOS in AMRS-induced anxiety during protracted EtOHW, another cohort of EtOHW rats was sequentially treated with 7-NI and SNP and then tested in the EPM (Figure 1). As shown in Figure 7, one-way ANOVA and posthoc tests showed that intra-NTS infusion of 7-NI once again blocked the AMRS-induced anxiety during EtOHW; however, subsequent administration of SNP abolished this anxiolytic effect [%entries_into open arms_: *F*_(3, 20)_ = 15.87, *p* < 0.001; saline/vehicle/AMRS/vehicle group (*n* = 6) versus EtOH/vehicle/AMRS/vehicle group (*n* = 6), *p* < 0.001; EtOH/vehicle/AMRS/vehicle group versus EtOH/7-NI/AMRS/vehicle group (*n* = 6), *p* < 0.001; EtOH/7-NI/AMRS/vehicle group versus EtOH/7-NI/AMRS/SNP group (*n* = 6), *p* < 0.01; saline/vehicle/AMRS/vehicle group versus EtOH/7-NI/AMRS/SNP group, *p* < 0.001; %time_spent in open arms_: *F*_(3, 20)_ = 11.17, *p* < 0.001; saline/vehicle/AMRS/vehicle group versus EtOH/vehicle/AMRS/vehicle group, *p* < 0.001; EtOH/vehicle/AMRS/vehicle group versus EtOH/7-NI/AMRS/vehicle group, *p* < 0.01; EtOH/7-NI/AMRS/vehicle group versus EtOH/7-NI/AMRS/SNP group, *p* < 0.05; saline/vehicle/AMRS/vehicle group versus EtOH/7-NI/AMRS/SNP group, *p* < 0.01].

## 4. Discussion

The results of the present study showed that 7-minute AMRS provoked anxiety-like behaviors, enhanced plasma CORT secretion, and sensitized NE release in the vBNST in rats treated with EtOH but not saline, at 28 days after the final dose of EtOH or saline. However, all of these behavioral, hormonal, and neurochemical abnormalities were attenuated by prior intra-NTS infusion of carboxy-PTIO, L-NAME, or 7-NI, but not by L-NIO. EtOHW elevated nNOS, but not eNOS, protein expression in the NTS, concomitant with an increased nNOS mRNA level, and the AMRS increased the phosphorylation rate of nNOS in the NTS of EtOHW rats. Moreover, intra-NTS injection of SNP after 7-NI administration abolished the expected anxiolytic action of 7-NI. Taken together, these results suggest a critical role of solitary nNOS in anxiety and vBNST NE release induced by acute mild stress during protracted EtOHW.

The susceptibility to stress during protracted EtOHW alters neurotransmission responses to certain stimuli that are normally innocuous to provoke pathophysiological consequences [2, 27]. To mimic this, in the present study, rats were subjected to 7-minute AMRS at 28 days after cessation of EtOH when spontaneous EtOHW anxiety-like symptoms had disappeared. Similar to the results from Valdez et al. [11], the AMRS produced anxiety in the EtOHW rats but not in their saline-treated counterparts. These results also were in agreement with the report by Ostroumov et al. [28] indicating that restraint stress increased EtOH self-administration via altered tegmental GABA signaling. However, in the present study, the anxiety was blocked by prior intra-NTS injection of carboxy-PTIO, L-NAME, or 7-NI, but not L-NIO. These results were consistent with the reported anxiolytic effects of NOS inhibitors [18, 19] and compatible with the reports of Bonassoli et al. [22] and Gonzaga et al. [23] revealing that infusion of a nonselective NOS inhibitor, a selective nNOS inhibitor, or a selective inducible NOS (iNOS) inhibitor (N-([3-(aminomethyl)phenyl]methyl) ethanimidamide dihydrochloride) into the dorsolateral periaqueductal gray matter or into the dorsal raphe nucleus, respectively, attenuated EtOHW anxiety in rats 24 or 48 hours after discontinuation of EtOH. The expression of iNOS is induced in response to inflammatory and immune stimuli [29], and chronic EtOH may cause dysregulation of the immune system in the brain that can persist over a certain withdrawal time, while increased production of proinflammatory factors has shown to sensitize EtOHW anxiety [30–32]. However, in the present study, we did not examine the involvement of solitary iNOS, since it was not detected in the NTS of the protracted EtOHW rats in a preliminary experiment. Moreover, in the present study, the anxiolytic effects of the above mentioned agents were hormonally supported because ELISA showed that carboxy-PTIO, L-NAME, and 7-NI, but not L-NIO, attenuated the increase in plasma CORT secretion induced by AMRS during EtOHW. These results collectively suggest that solitary NO signaling via nNOS contributes to the anxiety induced by acute mild stress during protracted EtOHW.

Both anxiety and plasma CORT secretion are closely associated with increased noradrenergic transmission in the BNST [12, 33]. In the present study, in vivo microdialysis showed that AMRS evoked sensitized NE release in the vBNST of EtOHW rats but not EtOH-naive rats, which is in line with the finding by Schmidt et al. [34] showing that mice experiencing repeated restraint stress exhibited elevated NE release in the BNST across multiple optogenetic stimulation parameters compared with stress-naive mice. However, pretreatment with carboxy-PTIO, L-NAME, or 7-NI, but not L-NIO, prevented the increase in the extracellular NE level. These results were consistent with the behavioral and hormonal findings in the present study and provide direct evidence of heightened NE release in the vBNST by stress during EtOHW. The increased NE release in the vBNST arises from noradrenergic excitation of both the soma region (NTS-A2) and the terminal area (vBNST) [24, 34]. Nevertheless, in the present study, the rate of inhibition of NE release by intra-NTS carboxy-PTIO was ~80%, indicating that a large fraction of NE release was driven by excitation of NTS-A2 via NO signaling when challenged by AMRS during protracted EtOHW. These results suggest that inhibition of solitary NO signaling can attenuate AMRS-precipitated vBNST NE release, thereby mitigating anxiety during EtOHW.

The NTS primarily integrates and transmits visceral and external information to the forebrain, forming the autonomic-affective functional basis for the body. The gaseous molecule NO serves as both a neurotransmitter and neuromodulator, and is synthesized by three isoforms of NOS, i.e., nNOS, iNOS, and eNOS. We reported previously that systemic nicotine administration increased hypothalamic NE release via activation of both nNOS and eNOS in the NTS [25]. However, in the present study, L-NIO influenced neither anxiety nor NE release, which is consistent with the Western blot results that the EtOHW unaffected both eNOS and phospho-eNOS expressions in the NTS. By comparison, in the present study, both protein and mRNA levels of the solitary nNOS were increased during EtOHW; in addition, the solitary phospho-nNOS protein levels were significantly increased in EtOHW/AMRS rats but neither in EtOHW/non-AMRS rats nor in saline/AMRS rats. These results indicate that the nNOS system in the NTS during protracted EtOHW is in its “elevated response mode toward stress” and highly activated when challenged by AMRS, which sensitizes behavioral and noradrenergic response. This idea is also supported in the present study by the behavioral result that subsequent administration of SNP into the NTS abrogated the anxiolytic effect of 7-NI. Taken together, these findings reinforce the suggestion that solitary nNOS, but not eNOS, mediates the sensitized behavioral and neurochemical responses to AMRS during EtOHW. nNOS and eNOS are expressed in different types of cells in the NTS [35] that have different functions. For example, nNOS-derived NO induces glutamate release [36], whereas eNOS-derived NO enhances GABAergic transmission [37]. Thus, although the precise reasons have yet to be elucidated, these anatomical and physiological distinctions may at least partly account for the different effects of EtOHW on solitary nNOS and eNOS.

**Figure 8:**
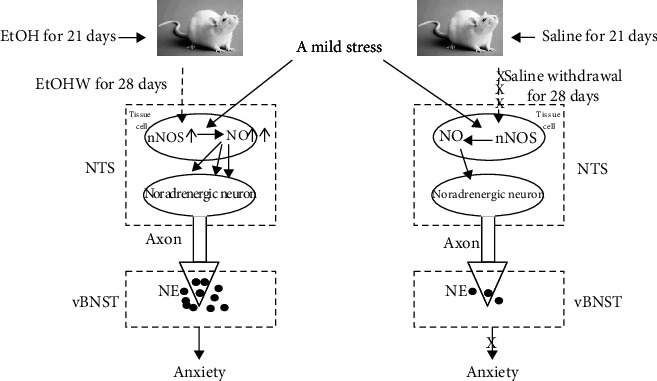
Mechanisms of AMRS-provoking anxiety during protracted EtOHW. EtOH: ethanol; EtOHW: EtOH withdrawal; NO: nitric oxide; nNOS: neuronal NO synthase; NE: norepinephrine; NTS: nucleus tractus solitarius; vBNST: ventral bed nucleus of the stria terminalis.

In summary, prior intra-NTS infusion of carboxy-PTIO, L-NAME, or 7-NI, but not L-NIO, attenuated the anxiety and vBNST NE release induced by 7-minute AMRS during EtOHW. EtOHW enhanced both nNOS protein and mRNA expression in the NTS but did not affect the eNOS protein level. These observations suggest that nNOS activity is promoted in the NTS during protracted EtOHW, which sensitizes the NTS-BNST noradrenergic response to stress and results in anxiety-like behavior in rats (Figure 8).

## Data Availability

The data supporting the conclusions in this study are statistically analyzed and included in Results section and are available from the corresponding author on reasonable request.
